# Increased Zinc Availability Enhances Initial Aggregation and Biofilm Formation of *Streptococcus pneumoniae*

**DOI:** 10.3389/fcimb.2017.00233

**Published:** 2017-06-07

**Authors:** Lindsey R. Brown, Rachel C. Caulkins, Tyler E. Schartel, Jason W. Rosch, Erin S. Honsa, Stacey Schultz-Cherry, Victoria A. Meliopoulos, Sean Cherry, Justin A. Thornton

**Affiliations:** ^1^Department of Biological Sciences, Mississippi State UniversityStarkville, MS, United States; ^2^Department of Infectious Diseases, St. Jude Children's Research HospitalMemphis, TN, United States

**Keywords:** zinc, pneumococcus, cell-cell interactions, biofilms, colonization

## Abstract

Bacteria growing within biofilms are protected from antibiotics and the immune system. Within these structures, horizontal transfer of genes encoding virulence factors, and promoting antibiotic resistance occurs, making biofilms an extremely important aspect of pneumococcal colonization and persistence. Identifying environmental cues that contribute to the formation of biofilms is critical to understanding pneumococcal colonization and infection. Iron has been shown to be essential for the formation of pneumococcal biofilms; however, the role of other physiologically important metals such as copper, zinc, and manganese has been largely neglected. In this study, we investigated the effect of metals on pneumococcal aggregation and early biofilm formation. Our results show that biofilms increase as zinc concentrations increase. The effect was found to be zinc-specific, as altering copper and manganese concentrations did not affect biofilm formation. Scanning electron microscopy analysis revealed structural differences between biofilms grown in varying concentrations of zinc. Analysis of biofilm formation in a mutant strain lacking the peroxide-generating enzyme pyruvate oxidase, SpxB, revealed that zinc does not protect against pneumococcal H_2_O_2_. Further, analysis of a mutant strain lacking the major autolysin, LytA, indicated the role of zinc as a negative regulator of LytA-dependent autolysis, which could affect biofilm formation. Additionally, analysis of cell-cell aggregation via plating and microscopy revealed that high concentrations of zinc contribute to intercellular interaction of pneumococci. The findings from this study demonstrate that metal availability contributes to the ability of pneumococci to form aggregates and subsequently, biofilms.

## Introduction

*Streptococcus pneumoniae*, or pneumococcus, asymptomatically colonizes the human nasopharynx and causes chronic otitis media through the formation of biofilms. Biofilms are highly structured bacterial communities that are protected from environmental stressors. The ability to form biofilms is known to contribute to persisting colonization and survival of pneumococcus on nasopharyngeal cells and within the middle ear of the host (Honsa et al., 2013). Colonization of the lung is also an important precursor for invasion into the bloodstream (Ogunniyi et al., 2010). It has been shown that bacteria found growing within biofilms are less virulent than those grown planktonically; however, pneumococcal aggregates have also been shown to form in the myocardium of the heart during invasive pneumococcal disease (Lizcano et al., 2010; Sanchez et al., 2011; Brown et al., 2014; Gilley and Orihuela, 2014; Brown and Orihuela, 2015). Pneumococcal biofilms are known to be regulated by a variety of factors including, quorum sensing molecules, choline availability, and exogenous iron (Moscoso et al., 2006; Trappetti et al., 2011a,b). Since pneumococcal biofilms are essential for both colonization and persistence, it is important to continue to investigate the environmental stressors that affect their formation.

Metal concentrations vary from host to host, and pneumococcus encounters additional changes in metals upon aspiration from the upper respiratory tract into the lungs or following transmigration into the blood or cerebrospinal fluid (McDevitt et al., 2011). Extensive research has aimed to identify how metals alter the organism's ability to cause invasive disease. Metals such as zinc, copper, manganese, and magnesium are required as cofactors and structural components of many bacterial proteins, including those of *S. pneumoniae*, and are known to be essential for proper metabolism and cellular defenses (Waldron et al., 2009; Honsa et al., 2013). Although there is vast knowledge on the importance of metals for bacterial cells, the effect of these metals on biofilm formation in the pneumococcus have been largely ignored.

Zinc is the second most abundant trace metal ion in the human body, and plays a role in a variety of cellular functions (Kehl-Fie and Skaar, 2010). Though zinc availability in the body ranges from 5 to 300 μM (Bayle et al., 2011), McDevitt et al. have found zinc concentrations surpassing 600 μM in the bloodstream of mice infected with *S. pneumoniae* (McDevitt et al., 2011). Pneumococcus acquires zinc from the extracellular environment by zinc transport proteins, AdcA and AdcAII, and the Pht proteins, PhtA, PhtB, PhtD, and PhtE (Plumptre et al., 2014a,b; Eijkelkamp et al., 2016). Mutants lacking the genes encoding AdcA and AdcAII were shown to be deficient in zinc uptake and cell growth; and less virulent in intranasal and intraperitoneal infection models (Bayle et al., 2011; Plumptre et al., 2014a; Brown et al., 2016). Additionally, a previous study from our laboratory showed the importance of AdcAII, specifically, to adhesion *in vitro* and colonization *in vivo*, thus suggesting a role for zinc homeostasis in colonization and biofilm development of *S. pneumoniae* (Brown et al., 2016). Interestingly, evidence shows the importance of zinc for surface protein interactions that contribute to aggregation and biofilm formation of *Staphylococcus aureus*, another significant human pathogen (Conrady et al., 2008; Formosa-Dague et al., 2016). However, in contrast, high concentrations of zinc have also been shown to inhibit biofilm formation in the Gram-positive organism *Streptococcus suis* (Wu et al., 2013).

Since biofilms are an integral component of colonization, we hypothesized that zinc availability will affect the initial stages of biofilm formation. Our approach was to observe early stage construction of pneumococcal biofilms in a broad range of physiologically relevant zinc concentrations. Here, we investigate the influence of zinc availability on cell-cell interactions, LytA-dependent autolysis, and initial biofilm formation. We have demonstrated that abundant zinc availability allows for the development of more substantial pneumococcal biofilms *in vitro*, modestly increases colonization *in vivo*, and delays the onset of autolysis. These results contribute to our understanding of the role of zinc in pneumococcal aggregation and biofilm formation, suggesting a potential target for antimicrobials that aim to reduce bacterial burdens of *S. pneumoniae* and potentially other Gram-positive organisms.

## Materials and methods

### Ethical statement

All animal studies were performed in compliance with a protocol reviewed and approved by the Institutional Animal Care and Use Committee at Mississippi State University (IACUC protocol #14-016). Animal husbandry was provided by veterinary staff and technicians within the Association for Assessment and Accreditation of Laboratory Animal Care and the National Institutes of Health Office of Laboratory Animal Welfare assured program at MSU. All work was performed in adherence to the United States Public Health Service Policy on Humane Care and Use of Laboratory Animals and Guide for the Care and Use of Laboratory Animals. Additionally, all experiments were performed in accordance with protocols approved by the Mississippi State University Institutional Biosafety Committee (IBC protocol #004-16).

### Bacterial strains and growth conditions

These studies utilized *S. pneumoniae* strains TIGR4, its unencapsulated mutant (T4R), and EF3030. All strains were grown on tryptic soy agar plates supplemented with 5% defibrinated sheep blood or in Todd-Hewitt broth (THB; BD Biosciences, Sparks MD; Tettelin et al., 2001; Fernebro et al., 2004). Bacterial strains were grown to an optical density at 600 nm (*OD*_600_) of 0.6 and stored at −80°C in 20% glycerol for later use. Chelex Resin (Bio Rad) was added to THB medium (5 g/100 mL) prior to autoclaving to chelate out all metals. THB + Chelex was then stirred overnight at room temperature, and sterile filtered the following day. For assays looking at the effect of specific metals, metals were added back to Chelex-treated THB at the following concentrations unless otherwise indicated: calcium (500 μM), copper (10 μM), iron (100 μM), magnesium (500 μM), manganese (100 μM), and 100 μM zinc (100 μM). Mutants of T4R lacking SpxB (ΔSpxB) and LytA (ΔLytA) were created using splicing by overlap extension (SOE) PCR method using an erythromycin or spectinomycin antibiotic cassette and standard *S. pneumoniae* transformation procedures (Ho et al., 1989). Mutants lacking SpxB and LytA were isolated by selection on blood agar plates supplemented with 0.5 μg/mL erythromycin or 500 μg/mL spectinomycin and confirmed by PCR. Bacterial aliquots were subsequently stored at −80°C in the same media conditions. Quantitative RT-PCR was used to assess expression of *Sp_0741* and *Sp_1935*, genes downstream of SpxB and LytA, respectively, to verify that polar mutations were not present. Growth curves were performed by diluting bacteria to final concentration of 1 × 10^5^ CFU/mL into various media conditions, 200 μL were inoculated into 96-well-plates in triplicate. Plates were then read at *OD*_600_ every 30 min for 20 h. All growth curves were performed at least three times and figures display a representative curve for each strain.

### Biofilm assays

Assays were performed in 6-well-polystyrene plates. Bacteria were diluted into Chelex-treated THB to a final concentration of 1 × 10^5^ CFU/mL and 2 mL were added to 6-well-plates. Plates were incubated for 6 h at 37°C with 5% CO_2_. Following incubation, plates were rinsed with 2 mL PBS. Biofilms were stained with 1 mL of 0.05% crystal violet for 10 min and rinsed twice with PBS. Biofilms were then air dried in a biosafety cabinet until dry and crystal violet was solubilized with 2 mL 95% ethanol. Plates were placed on a shake plate for 15 min, 200 μL ethanol was transferred to a sterile 96-well-plate in triplicate, and plates were read in a spectrophotometer at *OD*_540nm_. For viability plating of biofilms, plates were inoculated in the same manner as for crystal violet staining; however, following incubation, non-biofilm culture media was removed; and biofilms were resuspended in 1 mL PBS and mechanically disrupted using cell scrapers. All samples were then serially diluted and plated on tryptic soy agar with 5% sheep blood, incubated overnight at 37°C with 5% CO_2_, and colonies were enumerated the following day. Planktonic growth was assessed by performing the same dilutions as those used for biofilm assay; however, cultures were transferred to glass culture tubes and incubated for 6 h at 37°C. Following incubation, serial dilutions were performed and plated on blood agar plates, and incubated overnight at 37°C with 5% CO_2_.

### Animal challenge

Female C57/BL6 mice were anesthetized and challenged intranasally with ~2 × 10^5^ CFU/10 μL of *S. pneumoniae* EF3030 grown in Chelex-treated THB supplemented with either 0 or 250 μM zinc chloride. On day 3 post-inoculation, animals were either humanely euthanized, or given an intranasal booster of 20 μL PBS containing 0 or 250 μM zinc chloride. On day 6 post-inoculation, the remaining animals were humanely euthanized by deep isoflurane inhalation followed by cervical dislocation and confirmation by incubation under CO_2_. Nasal washes and nasal tissues were collected from all animals by previously described methods, with the modification of using PBS instead of Ringer's lactate solution (Keller et al., 2015). Both nasal washes and homogenized tissues were serially diluted and plated on blood agar containing 20 μg/mL neomycin to inhibit growth of *Staphylococcus*. Following incubation, colonies from nasal washes and nasal tissues were enumerated and combined to represent a total CFU count of pneumococci colonizing the nasopharynx. Additionally, nasal tissue homogenates were centrifuged for 600 rpm for 3 min using a Shandon Cytospin 2. Samples were stained using a neat stain hemotology stain kit (Electron Microscopy Services #27104-01) and tissues were imaged using a Zeiss Axioskop 2 Plus.

### Primary human bronchotracheal cells

NHBE cells (Lonza, Walkersville, MD, USA) were plated in 0.33 cm^2^ transwell inserts (Corning, Corning, NY, USA) at a seeding density of 1.5 × 10^4^ cells per well and maintained in culture as previously described (Krunkosky et al., 2003). Briefly, cells were maintained in bronchial epithelial basal media (BEBM, Lonza) supplemented with the bronchial epithelial growth media (BEGM) BulletKit (Lonza) until confluent. An air/liquid interface (ALI) was established where the apical surface was exposed to a humidified 95% air/5% CO_2_ environment. Medium in the basolateral compartment was changed to ALI media (1:1 DMEM:BEBM) supplemented with BEGM Bullet Kit. The basolateral medium was changed every 2 days for a minimum of 5 weeks in culture to allow the cells to differentiate. For biofilm experiments, parental T4R (1 × 10^5^/10 μL) were inoculated into the apical chamber directly into the mucous layer. Medium in the basolateral chamber was replaced with media containing 0, 100, and 500 μM zinc. Following 24 h incubation, transwell inserts were then prepped for SEM.

### Scanning electron microscopy

Biofilms were grown as described above in 6-well-plates on Thermanox coverslips (Thermo Scientific #174934) for 6 h or on the apical surface of primary human epithelial cells for 24 h at 37°C with 0.5% CO_2_. Following incubation, biofilms were fixed with half-strength Karnovsky's fixative (3% glutaraldehyde and 2% paraformaldehyde) overnight at 4°C. Samples were then rinsed four times with deionized water before 1 h fixation with 0.1 M osmium tetroxide. Samples were rinsed with diH_2_O and dehydrated using a gradient of ethanol (30–100%). Samples were then chemically dried using a gradient of hexamethyldisalzone (HMDS) and air-dried overnight prior to mounting and sputter coating with 15 nm platinum. Biofilms were imaged using a JEOL 6500F Field Emission SEM.

### Aggregation assays

In order to assess aggregation of cells by plating, a 5 mL culture of T4R was grown in C+Y medium to 0.7 *OD*_600nm_. The culture was separated into three 1.5 mL cultures with each one receiving 0, 100, or 500 μM zinc, respectively. Samples were incubated on ice for 1 and 2 h. At each time point, samples were vortexed, serially diluted, and plated. In order to view cell aggregation by crystal violet staining, a 5 mL culture of T4R was grown to 0.7 *OD*_600nm_, 3-1 mL cultures were centrifuged at 13,000 rpm for 5 min and pellets were resuspended in PBS with 0, 100, or 500 μM zinc. One milliliter of each culture was added to a chamber well slide and incubated on ice for 1 h. Following incubation, PBS was removed and slides were stained with 0.05% crystal violet for 10 min, rinsed, and imaged at 20× magnification using a Zeiss Axioskop 2 Plus.

### Fluorescence microscopy

Aggregation assays were performed as described above. After 1 h on ice, chamber wells were rinsed with PBS and fixed with 4% paraformaldehyde (in PBS) for 10 min at 25°C. Wells were then rinsed with PBS and blocked with PBS plus 0.5% BSA and 4% mouse serum for 30 min at 25°C. Samples were then incubated with anti-TEPC-15 primary antibody (Sigma-Aldrich M-1421, specific for cell wall phosphorylcholine at 1:40) overnight at 4°C, rinsed two times with PBS for 5 min each, prior to being incubated with goat anti-mouse IgA conjugated to rhodamine (Southern Biotech 1040-03 Birmingham, AL, at 1:100) for 1 h. Wells were then rinsed with PBS, chambers were removed, 10 μL of VECTASHIELD Mounting Medium (Vector H-1000) was added, and a cover slip was applied. Samples were imaged at 40× magnification by fluorescence microscopy using a Zeiss Axioskop 2 Plus.

### Autolysis assays

*S. pneumoniae* cultures of T4R and ΔLytA were grown in 6 mL Chelex-treated THB in identical metal concentrations used for biofilm assays. Upon reaching *OD*_600nm_ of 0.5, cultures were divided into two 3 mL cultures. All culture tubes were centrifuged at 13,000 rpm for 5 min and pellets were resuspended in 3 mL Chelex-treated THB without added metals. Cultures were then treated with a final concentration of 0.05% sodium deoxycholate to induce autolysis. Control cultures received 40 μL water in place of detergent. Optical density (*OD*_600nm_) was recorded every 60 s for a total of 10 min. Optical densities at each time point were subtracted from the starting *OD*_600nm_) and plotted on an inverted scale to represent the amount of autolysis occurring over the 10 min timeframe.

### Statistical analyses

Biofilm experiments were repeated at least three times, results from independent experiments were averaged together and standard error of the mean was calculated. Data were analyzed only across concentrations that did not affect growth using a non-parametric Kruskal Wallis test. Animal challenge data are presented as the sum of CFU counts collected from nasal washes and nasal tissues collected from animals from two separate experiments. The data collected from the animal challenge, both colony and neutrophil counts were analyzed using a non-parametric Mann Whitney *U*-test, comparing animals from the 0 μM treatment group to the 250 μM treatment group. Aggregation assays by plating were repeated three times and CFU counts were averaged together. Data from those assays were analyzed using a Mann Whitney *U*-test comparing the 100 and 500 μM against the 0 μM control sample. All analyses were conducted with alpha = 0.05. Results were considered statistically significant when *p* < alpha. All statistical analyses were performed using GraphPad Prism 7.

## Results

### Zinc availability enhances biofilm formation

**Figure 1:**
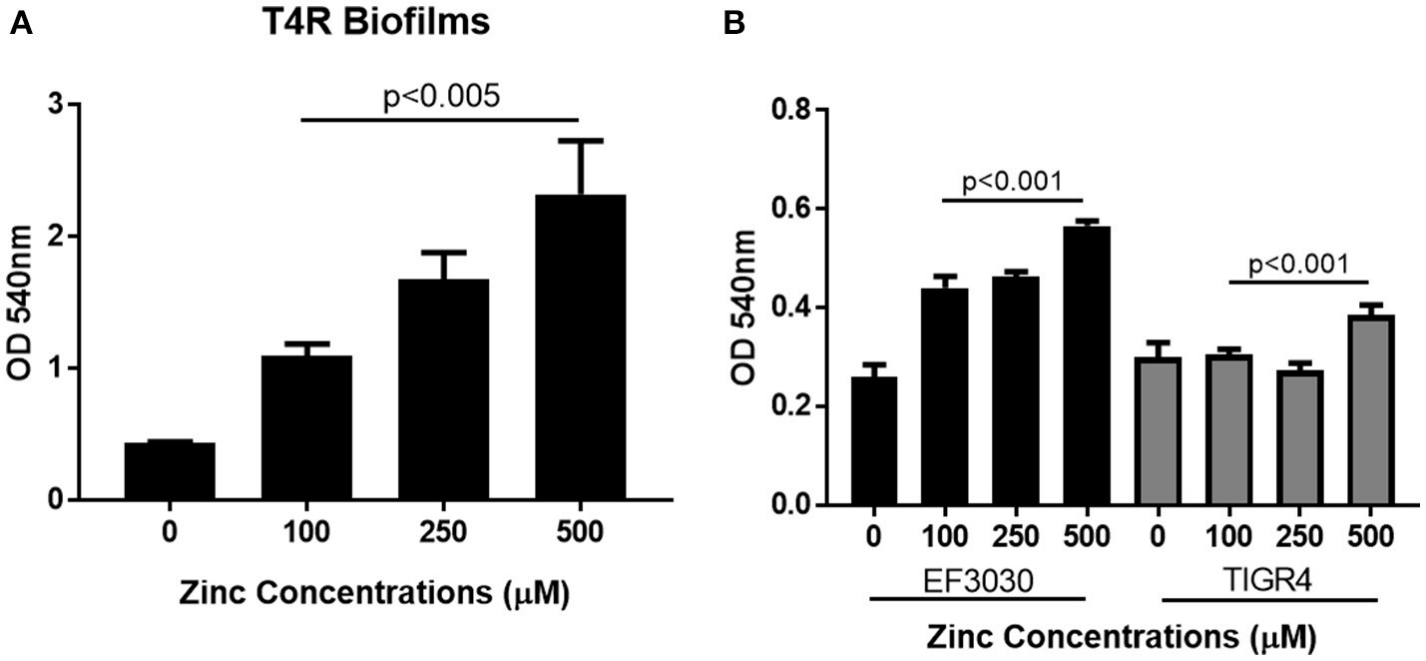
Zinc availability enhances biofilm formation. Biofilms increase in **(A)** unencapsulated T4R and **(B)** encapsulated strains EF3030 and TIGR4, as zinc availability increases in a dose dependent manner. Biofilms were grown in Chelex-treated THB pH 6.5 with metals supplemented, and zinc ranging from 0 to 500 μM. Following 6 h incubation, biofilms were rinsed and stained with crystal violet. Kruskal Wallis analysis comparing the mean *OD*_600nm_ from 100, 250, and 500 μM concentrations indicated a significance level of *p* < 0.001 for both TIGR4 and EF3030, and *p* < 0.005 for the T4R strain.

**Figure 2:**
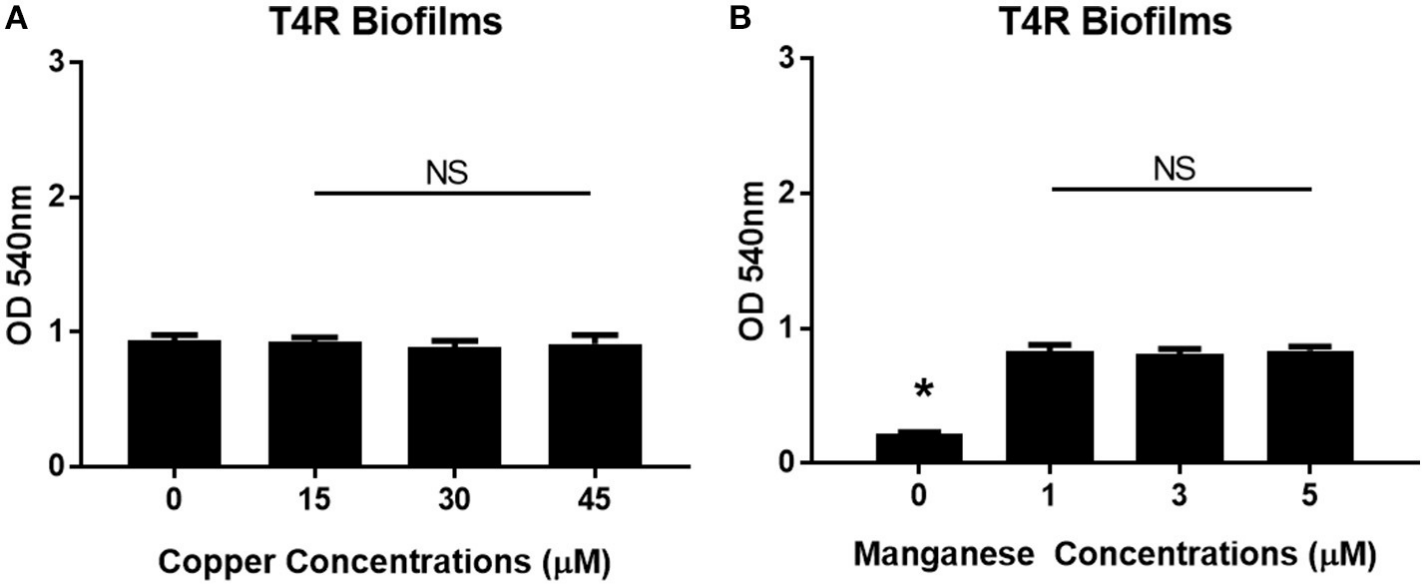
Increased biofilm phenotype is zinc-dependent. Biofilms grown in Chelex-treated THB pH 6.5 with varying concentrations of **(A)** copper (0–45 μM) and **(B)** manganese (0–5 μM). Kruskal Wallis analysis comparing the mean of each concentration and indicated no statistical significance with *p* = 0.81 and *p* = 0.85, for copper and manganese, respectively. Sample exposed to 0 μM manganese failed to grow as indicated by (*).

Previous work has shown that zinc homeostasis is essential for growth of pneumococcus, as mutants lacking both zinc-binding lipoproteins AdcA and AdcAII show decreased ability to grow in zinc-limited environments (Plumptre et al., 2014a); and mutants lacking only AdcAII were significantly less able to colonize the murine nasopharynx (Brown et al., 2016). Biofilms of *S. pneumoniae* strains T4R, TIGR4, and EF3030 were grown for 6 h in Chelex-treated THB supplemented with metals, and zinc supplemented at a wide range of physiologically relevant concentrations (0–500 μM). These strains were chosen to represent both invasive and colonizing strains of pneumococci. TIGR4 is an invasive strain originally isolated from the blood of a 30 year old patient, T4R is an unencapsulated mutant of TIGR4 that forms more efficient biofilms, and the colonizing strain EF3030 does not result in invasive disease (Briles et al., 1992; Aaberge et al., 1995; Tettelin et al., 2001). As zinc concentrations increased, early biofilm formation (6 h) increased in a dose-dependent manner in both the T4R and EF3030 strains (Figures 1A,B). In the TIGR4 strain, an increase in biofilm density was only detected in 500 μM zinc, while a slight decrease was seen in the 250 μM concentration, though this was not statistically different than the biofilms produced in 100 μM zinc (Figure 1B). The difference seen in TIGR4 could potentially be attributed to differences in biofilm production (Figure 1B). To determine if the effect on biofilm formation was zinc-specific, the same experiments were done manipulating both copper and manganese, utilizing lower concentrations that more closely mimic the range of these metals that pneumococcus would encounter during infection (McDevitt et al., 2011). Interestingly, altering copper (Figure 2A) and manganese (Figure 2B) had no effect on biofilm formation, apart from biofilms receiving 0 μM manganese, though this was due to an inability to grow without manganese supplementation. Since, AdcAII has been shown to contribute to adhesion and colonization, biofilm formation was assessed in an ΔAdcAII mutant. Results from this assay were similar to those of the parental T4R strain, suggesting AdcAII is not involved in pneumococcal biofilm formation (Supplemental Figure 1). In all conditions tested for the T4R strain, lack of biofilms were seen in samples treated with 0 μM metal, which was attributed to reduced ability to grow, as supplementation of metals is essential to bacterial survival (Supplemental Figure 2). As such, only biofilm samples that received metal and did not have altered growth were included in statistical analyses, indicated in the figures by a horizontal bar.

**Figure 3:**
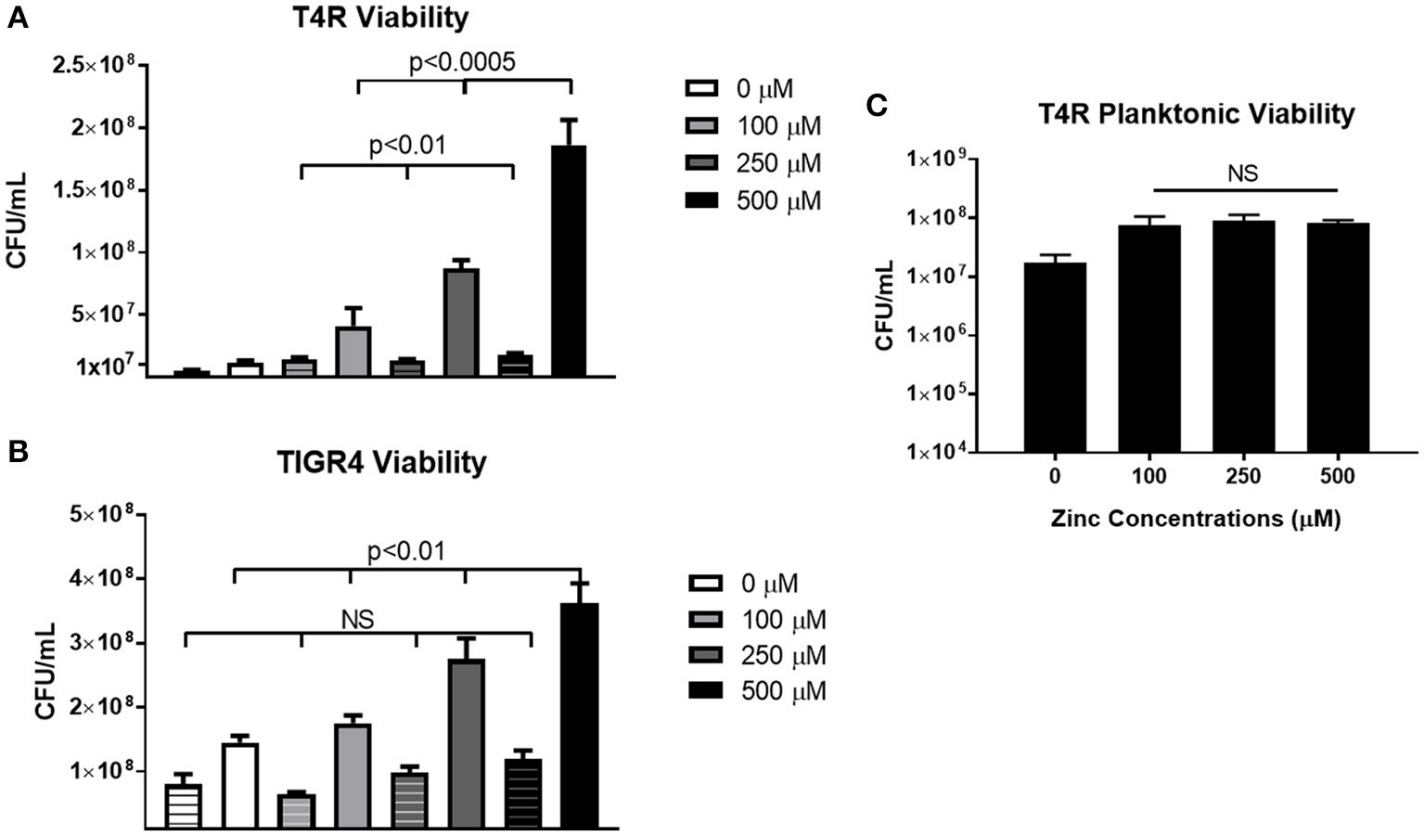
Viability of *S. pneumoniae* treated with 0–500 μM zinc. **(A)** Plate counts of T4R representing cells within both the non-biofilm culture media (indicated by striped bars) and biofilm (indicated by solid bars). Kruskal Wallis analysis indicated *p* < 0.0005 for non-biofilm culture media samples, and *p* < 0.01 for biofilm samples in the T4R strain. **(B)** Counts from both non-biofilm culture media (striped bars) and biofilm cells (solid bars) of the TIGR4 strain revealed a significance level of *p* < 0.01 in the biofilm samples, and no statistical significance in the non-biofilm culture media samples following Kruskal Wallis analysis. **(C)** Viability of T4R grown planktonically in culture tubes with varying concentrations of zinc (100–500 μM) revealed no statistical differences following Kruskal Wallis analysis, *p* = 0.89.

In order to determine how cell viability contributed to biofilm density, both non-biofilm culture media and biofilms were collected and plated following the 6 h incubation period. In the T4R strain, significant differences were seen in the total number of viable cells found within both the supernatant and biofilms as zinc availability increased (Figure 3A). Interestingly though, only biofilm viability was significantly different across the treatment concentrations in the TIGR4 biofilms (Figure 3B). Additionally, there were no significant differences in viability among the 100–500 μM zinc concentrations in both the T4R and EF3030 cultures grown planktonically in glass culture tubes (Figure 3C and Supplemental Figure 3). Surprisingly, though no decrease in viability was seen in 500 μM TIGR4 biofilms samples, cultures grown planktonically showed a significant decrease in viability when grown in the presence of 500 μM zinc (Supplemental Figure 3). These data indicate that zinc does not appear to strongly enhance growth, and therefore cannot be the primary factor contributing to the phenotype shown in Figure 1; moreover, zinc could potentially be contributing to cell-cell interactions between pneumococci within biofilm structures.

**Figure 4:**
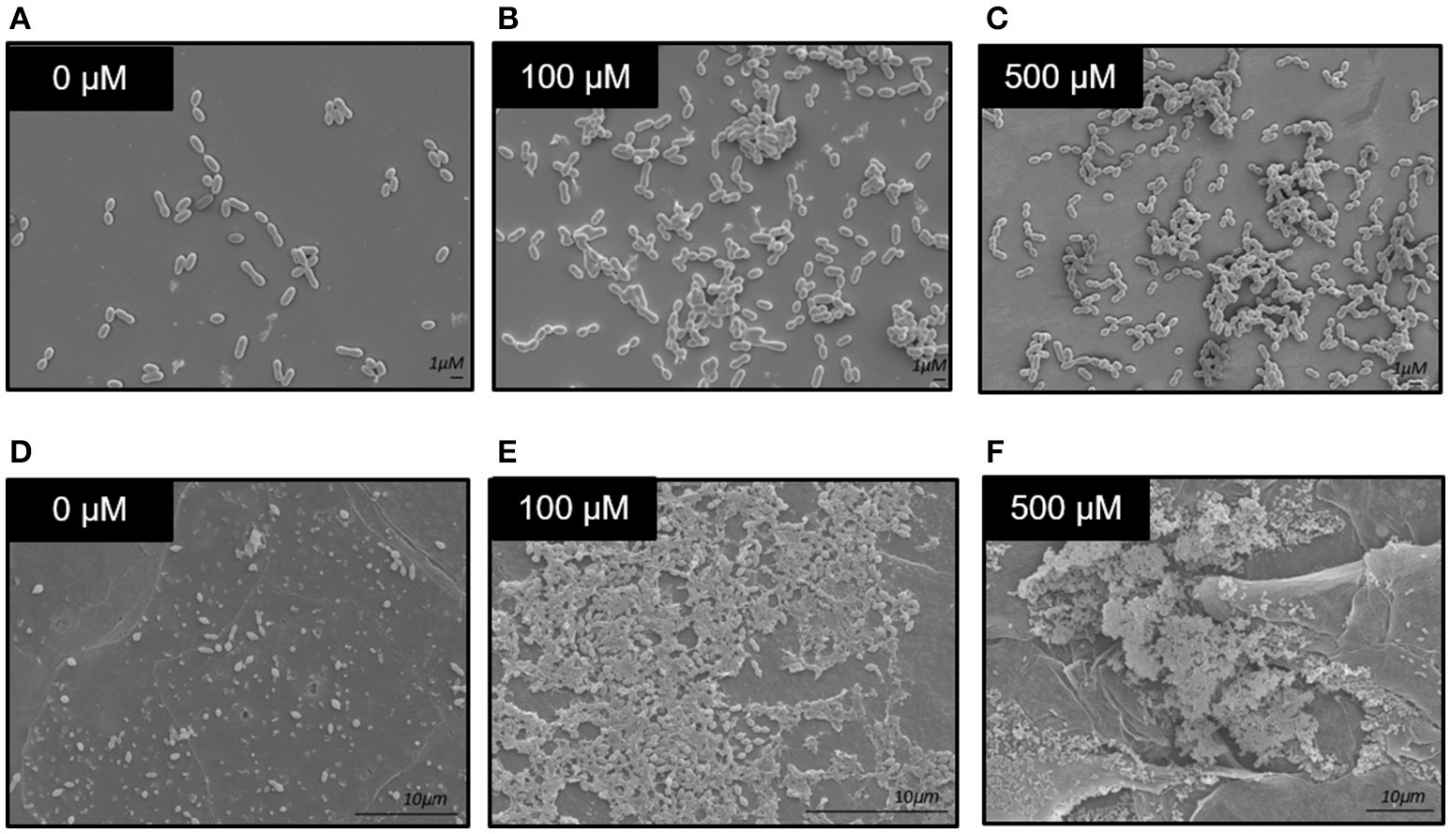
Scanning electron microscopy of *S. pneumoniae* biofilms. **(A–C)** Micrographs of 6 h biofilms grown in the presence of 0–500 μM zinc on Thermanox coverslips grown in 6-well polystyrene dishes. **(D–F)** Micrographs of 24 h biofilms grown in the presence of 0–500 μM zinc, on the surface of primary human bronchotracheal epithelial cells.

In addition to viability plating, scanning electron microscopy (SEM) was used to assess biofilms grown on Thermanox coverslips. Micrographs demonstrated that growth in the presence of greater zinc concentrations led to formation of more substantial bacterial clusters (Figures 4A–C). To investigate a more physiologically relevant model, T4R was inoculated into the apical chamber of transwells containing a monolayer of differentiated primary human bronchotracheal, and allowed to grow for 24 h in the presence of various concentrations of zinc. Following incubation, the cell monolayer was removed from the transwell and processed and analyzed via SEM. At the 24 h time point, the bacteria attached to the cell monolayer showed increasing aggregations as the zinc availability increased, similarly to what was seen in the previous static biofilm assays. Most interesting; however, were the large three-dimensional plaque-like structures formed by the bacteria in the samples treated with 500 μM zinc (Figures 4D–F).

### Zinc does not significantly alter *In vivo* colonization

**Figure 5:**
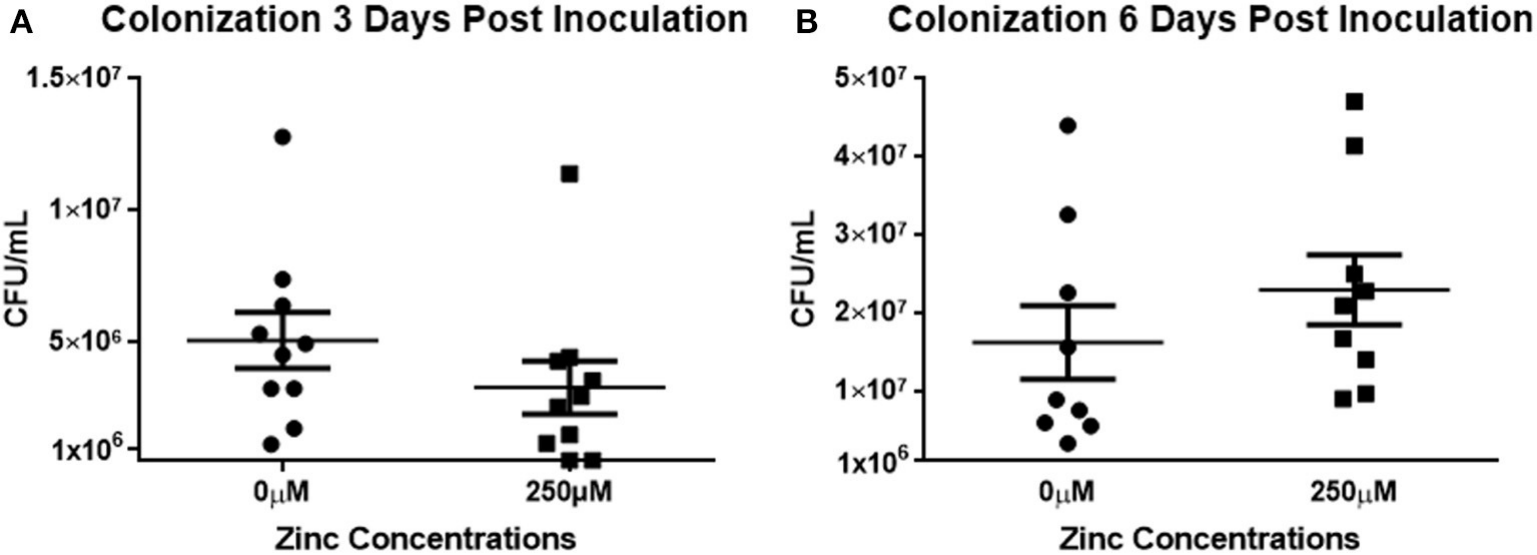
Zinc does not alter *in vivo* colonization. **(A)** Mice were inoculated intranasally with the colonizing strain of EF3030 grown in either 0 or 250 μM zinc (2 × 10^5^ CFU/10 μL). One group was sacrificed at day 3; a second group was treated with a zinc booster of 0 or 250 μM PBS 3 days post-inoculation. Six days post-inoculation, remaining mice were sacrificed. Nasal washes and tissues were collected from each animal, homogenized, serially diluted, and plated. Colony counts of nasal tissues and nasal washes were combined to determine representative values of pneumococci colonizing. Results were pooled from three independent experiments. Statistical analysis of colonization at **(A)** 3 days post-inoculation revealed *p* = 0.0853; whereas, analysis of colonization at **(B)** 6 days post-inoculation revealed *p* = 0.1359.

To determine if the effect of zinc on biofilms could be extended *in vivo*, mice were inoculated intranasally with *S. pneumoniae* EF3030 grown in 0 or 250 μM zinc and suspended in PBS containing 0 or 250 μM zinc. The EF3030 strain was utilized for this challenge due to its propensity for colonizing the respiratory tract rather than causing invasive disease (Briles et al., 1992 #103). At 3 days post-infection, mice received either a booster of 250 μM zinc or PBS alone; or were sacrificed to determine rates of colonization. At day 6 post-inoculation, colonization of the remaining animals was determined. Nasal washes and tissues were collected following euthanization of all animals involved in the challenge. At 3 days post-inoculation, colony forming units were modestly increased in the nasopharynx of the animals treated with the 0 μM inoculum compared to the 250 μM, though this did not reach statistical significance (Figure 5A). On the contrary, the data collected from the 6 day challenge indicated that the animals that received the 250 μM challenge inoculum had a slightly higher bacterial load compared to the animals from the 0 μM group, though again these data did not reach statistical significance (Figure 5B). Zinc has been shown to alter the neutrophilic response; therefore, upon conclusion of the study, nasal tissues were analyzed using histology staining and light microscopy to detect differences in neutrophil abundance between the treatment groups receiving 0 and 250 μM zinc boosters (Milanino et al., 1993; Freitas et al., 2009; Ong et al., 2015). No significant differences were detected in the number of neutrophils between the nasal tissues of the treatment groups, indicating no exacerbation in the inflammatory response (Supplemental Figure 4).

### Zinc does not function as an antioxidant in *S. pneumoniae* biofilms

Zinc has been shown to have similar antioxidant properties to manganese, in that it can antagonize redox-active metals such as iron or copper by limiting their production of reactive oxygen species (Powell, 2000). *S. pneumoniae* is known to produce up to millimolar concentrations of hydrogen peroxide as a byproduct of its metabolism by the enzyme pyruvate oxidase, SpxB (McLeod and Gordon, 1922; Pericone et al., 2003). Therefore, to determine if zinc functions as an antioxidant to protect against pneumococcal H_2_O_2_, biofilm formation was assessed in a mutant strain lacking SpxB (ΔSpxB). Since this strain produces trace amounts of hydrogen peroxide, if zinc were functioning as an antioxidant, there should be little difference in biofilm formation across the different zinc concentrations. Interestingly, the data from this assay demonstrated a similar dose-dependent effect to that of the parental T4R strain indicating no role for zinc in the protection against H_2_O_2_; however, a heightened sensitivity was seen in the ΔSpxB strain grown in 500 μM zinc compared to ΔSpxB grown in 0–250 μM concentrations (Supplemental Figure 5).

### Zinc regulates pneumococcal autolysis

**Figure 6:**
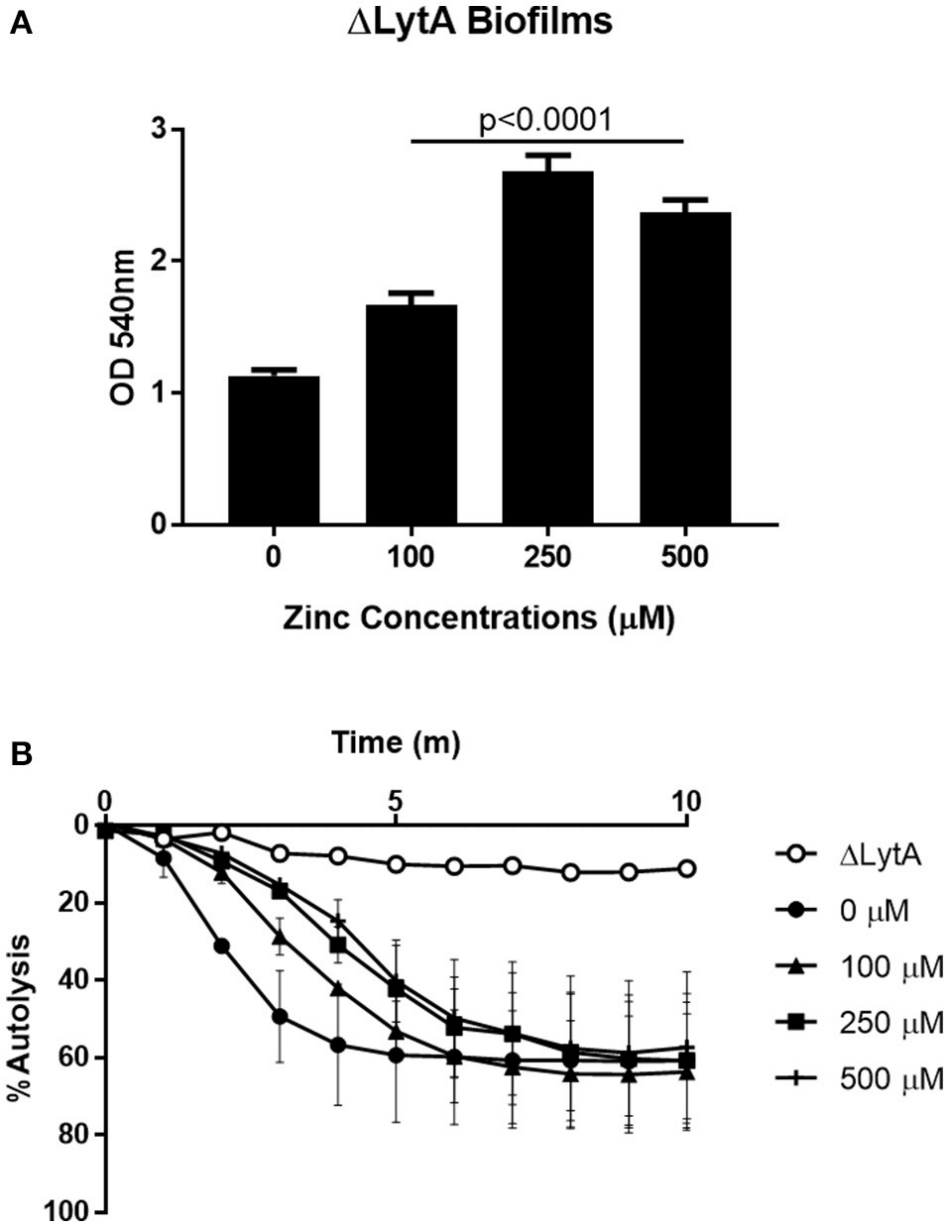
Zinc regulates pneumococcal autolysis. **(A)** Crystal violet staining of ΔLytA biofilms grown for 6 h in Chelex-treated THB pH 6.5 with metals supplemented and zinc supplemented at 0–500 μM. Kruskal Wallis analysis indicated a significance level of *p* < 0.0001 by comparing the means of samples treated with 100, 250, and 500 μM zinc. **(B)** T4R and ΔLytA cultures inoculated with DOC to induce autolysis, OD_600_ read every 60 s for 10 min. Autolysis curve shown is representative of three independent experiments.

The major pneumococcal autolysin, LytA, is known to contain an octahedrally coordinated zinc ion at the catalytic center, indicating that zinc is involved in the regulation of LytA (Mellroth et al., 2014). Pneumococcal autolysis can free DNA that could then contribute to biofilm formation as has been previously described (Steinmoen et al., 2002; Domenech et al., 2015). To determine if LytA is contributing to increased biofilm formation seen in the parental T4R strain, the effect of zinc on biofilm formation in a mutant strain lacking LytA (ΔLytA) was investigated. The loss of LytA did not eliminate the phenotype as shown in Figure 6A, and the mutant strain lacking LytA appeared more sensitive to the 500 μM zinc treatment (Supplemental Figure 6). These data indicate that the increase in biofilm density is not due to LytA-dependent DNA release. To determine if zinc was regulating LytA-dependent autolysis at physiologically relevant concentrations, similarly to what was shown by Höltje and Tomasz at 1–10 mM, cultures of T4R were grown in the presence of 0–500 μM zinc to an *OD*_600_ of 0.5 and treated with sodium deoxycholate (Höltje and Tomasz, 1976; Marriott et al., 2008; Figure 6B). Cultures grown in higher concentrations of zinc were more resistant DOC-induced autolysis, whereas, cultures grown without zinc completely autolysed within 5 min, a ΔLytA sample was included to show that the DOC-induced autolysis is LytA-dependent. This indicates that enhanced zinc availability protects against autolysis, and therefore is not likely enhancing autolysis-dependent DNA release that could in turn be contributing to biofilms.

### Zinc availability increases cell-cell aggregation

**Figure 7:**
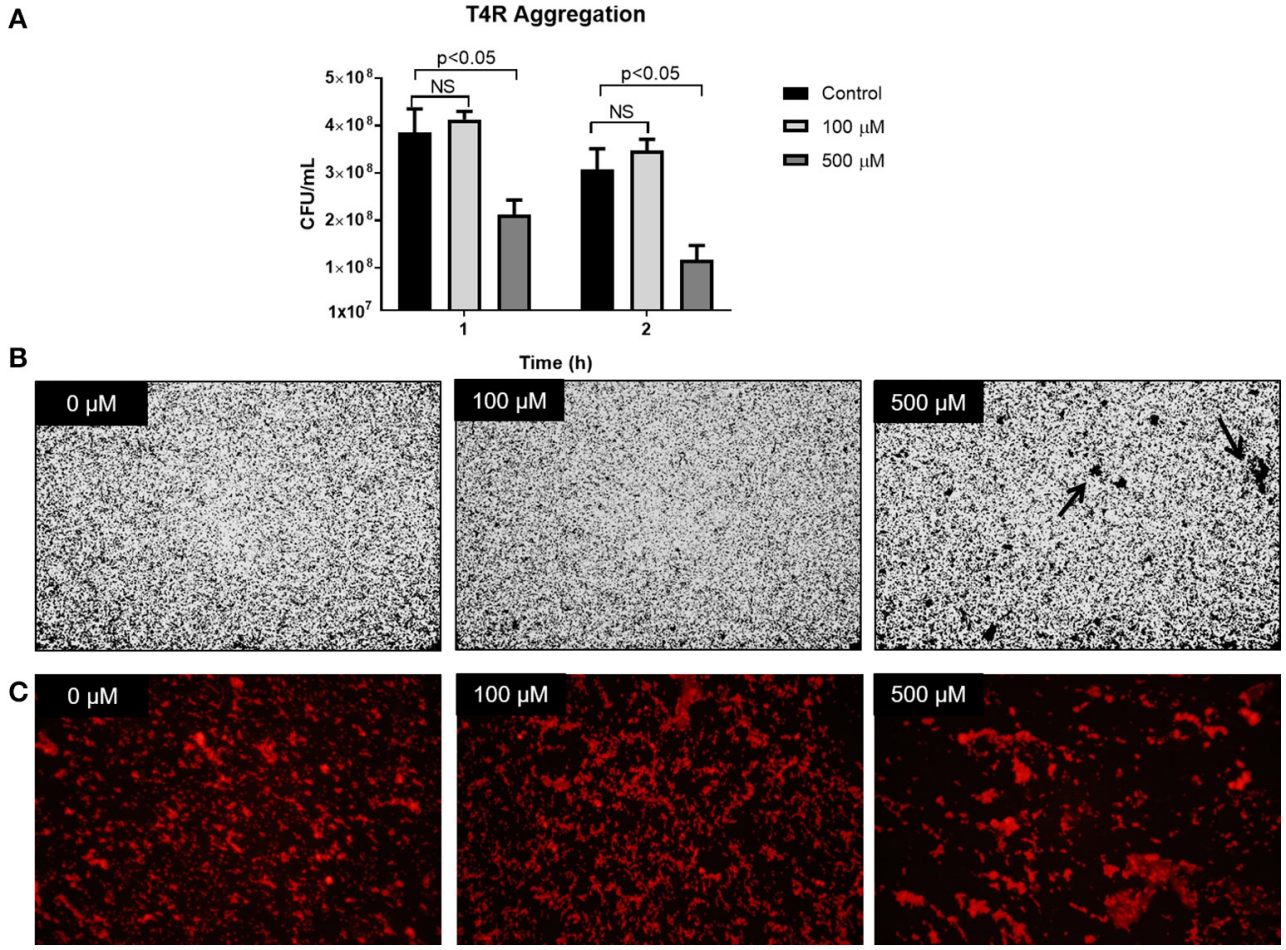
Zinc availability increases cell-cell aggregation. **(A)** T4R cultures were spiked with 0, 100, or 500 μM zinc, incubated on ice for 1 or 2 h, and plated for viability. Analysis via the non-parametric Mann-Whitney test revealed no significant difference between 0 and 100 μM samples at both the 1 and 2 h time points; however, analysis comparing the 0 and 500 μM samples revealed a significance level of *p* < 0.05 following both 1 and 2 h incubation on ice. **(B)** Light micrographs at 20x magnification of crystal violet stained T4R in chamber well slides following treatment with 0–500 μM zinc and 1 h incubation on ice. Noticeable aggregates (indicated by the black arrow) formed only in the samples treated with 500 μM zinc. **(C)** Fluorescent micrographs taken at 40x magnification of T4R aggregates formed after 1 h exposure to 0–500 μM zinc and 1 h incubation on ice.

To assess a possible role of zinc in cell-cell interactions, aggregation was assessed by both plating and microscopy. Cultures of T4R were grown to OD_600nm_ 0.7 and supplemented with either 100 or 500 μM zinc. Cultures were then incubated on ice to prevent growth of the cells for 1 and 2 h. Following incubation, the cultures were mixed, serially diluted, and plated. Results from this assay revealed that after 1 h in the presence of 500 μM zinc, the colony forming units within the culture were significantly diminished by nearly 50% (Figure 7A, *p* < 0.05). This phenotype was consistent in samples incubated on ice for 2 h. To verify the hypothesis of cell-cell clumping in the presence of excess zinc, similar assays were performed in which T4R was grown to OD_600nm_ 0.7 and 1 mL cultures were centrifuged and resuspended in PBS with 0, 100, or 500 μM zinc, transferred to a chamber well slide, and incubated on ice for 1 h. Following incubation on ice, wells were rinsed, stained with crystal violet, and imaged by light microscopy. Similarly, samples were fixed with paraformaldehyde, blocked with PBS containing BSA and mouse serum, incubated with a primary anti-cell wall phosphorylcholine antibody, incubated with a secondary goat anti-mouse IgA antibody tagged with rhodamine, and imaged by fluorescence microscopy. The hypothesis of zinc contributing to cell-cell aggregations was confirmed by the large clumps (indicated with an arrow) found present only in the sample treated with 500 μM zinc (Figure 7B). This phenotype was consistent in the fluorescent micrographs of samples treated with 500 μM zinc (Figure 7C). These data combined with the viability data shown in Figure 3 indicate that the phenotype consistently seen across the three *S. pneumoniae* strains tested is due to zinc increasing intercellular interactions at high concentrations.

## Discussion

*S. pneumoniae* is an obligate human pathogen commonly known for causing the invasive diseases: pneumonia, septicemia, and meningitis. The mechanisms of pneumococcal invasive diseases have been well-studied; however, in order for pneumococcus to disseminate and cause invasive disease it must first colonize the human nasopharynx. More than 1.5 million sinusitis and otitis media infections have resulted solely from pneumococcal colonization and biofilm formation, and in the United States alone, resulted in the spending of more than $927 million in treatment costs (Huang et al., 2011). Though pneumococci found primarily growing within biofilms have been shown to not be highly invasive, the formation of biofilms is critical for the epidemiology of this pathogen, along with many others (Jefferson, 2004; Hall-Stoodley and Stoodley, 2009; Sanchez et al., 2011; Gilley and Orihuela, 2014).

Zinc availability ranges within the respiratory tract of the human body from concentrations as low as 5 μM to as high as 300 μM (Bayle et al., 2011; McDevitt et al., 2011; Honsa et al., 2013). Zinc has been shown to regulate a variety of cellular functions in pneumococcus, including transcriptional regulation through the zinc-dependent repressor AdcR and manganese limitation through competitive binding of proteins involved in metal acquisition (Reyes-Caballero et al., 2010; McDevitt et al., 2011; Shafeeq et al., 2011; Eijkelkamp et al., 2014). Available zinc is known to be concentrated in certain microniches within the human body, one of which is the airway epithelium of the nasopharynx (Zalewski et al., 2005), We have previously shown that mutants lacking AdcAII, a zinc-binding surface protein of pneumococcus, are deficient in the ability to colonize the murine nasopharynx compared to wildtype TIGR4 (Brown et al., 2016). However, a role for zinc specifically in the formation of biofilms has, to our knowledge, not been described. In this study, we have demonstrated an additional aspect of pneumococcal pathophysiology that is sensitive to metal availability. Our results indicate that pneumococci that have encountered high concentrations of zinc, such as that found during neutrophilic response or during pneumococcal infection in the blood are capable of forming larger aggregates and biofilms within very short periods of time *in vitro*. Interestingly, we have shown that this is a zinc-dependent affect as altering copper or manganese led to virtually no change in biofilm formation. To determine if this phenotype extended into an animal model, we analyzed intranasal colonization in animals treated with 0 or 250 μM zinc. Animals in the high zinc treatment group were challenged with 250 μM as previous studies have shown that zinc inhalation can damage the olfactory system and result in anosmia as well as increased macrophages and lymphocytes in the lungs (Marrs et al., 1988; Duncan-Lewis et al., 2011). Though modest differences were seen between the two treatment groups, data collected from both 3 and 6 days post-inoculation did not reach statistical significance. We believe this could be due to high variance normally associated with *in vivo* colonization studies. Another limitation to this *in vivo* study could be the potential for zinc to alter the host immune response. In order to mitigate this, we assessed neutrophil abundance and saw no significant differences between the neutrophils present at the 3 and 6 day time points. It would be interesting to further investigate the effect of zinc on neutrophils at an earlier time point and potentially neutrophil recruitment.

Since zinc is known to play a role in protection against oxidative stress, we also investigated biofilm formation in mutants lacking SpxB, the protein responsible for the production of copious amounts of hydrogen peroxide. We expected that if the increase in biofilms we have observed is dependent on zinc protecting against H_2_O_2_, then there would be no difference in biofilm formation in mutants that produce trace amounts of H_2_O_2_. It appears that, in our model, zinc does not to protect against pneumococcal H_2_O_2_, though we did note that the ΔSpxB strain was more sensitive to the 500 μM zinc treatment than the parental T4R strain. We hypothesize this could be due to the fact that SpxB is critical for normal metabolic reactions to occur. A recent study from Ong et al. showed that zinc disrupts carbon metabolism in the closely related *Streptococcus pyogenes* (Ong et al., 2015). We are currently investigating the effect of zinc intoxication on metabolism in pneumococcal biofilms.

Extracellular DNA has been extensively characterized as playing a role in the formation of biofilms in several different bacterial species (Carrolo et al., 2010; Gloag et al., 2013; Tang et al., 2013). Pneumococcus specifically has the ability to release extracellular DNA through the process of LytA-dependent autolysis (Steinmoen et al., 2002; Domenech et al., 2015). Domenech et al. have published multiple studies highlighting the relationship between the choline-binding family of proteins and extracellular DNA in pneumococcal biofilms (Moscoso et al., 2006; Domenech et al., 2015). In order to investigate if LytA-dependent autolysis was contributing to our increasing biofilms in different concentrations of zinc, we utilized a mutant lacking LytA. However, the results from this mutant indicate that the effect of zinc on biofilm formation is not entirely LytA-dependent. We are also currently interested in the role of LytB and LytC as they have also been shown to interact with extracellular DNA in biofilm matrices (Domenech et al., 2015). Due to the presence of a zinc ion catalytic center at the heart of LytA, we investigated the effect of zinc supplementation on pneumococcal autolysis. The findings from these assays show that higher concentrations delay the onset of pneumococcal autolysis. These data indicate that zinc could potentially inhibit LytA-dependent autolysis which, rather than liberating intracellular DNA, could instead result in greater biomass, and thus contribute to biofilm formation.

Studies in *Staphylococcus aureus* have shown a role for zinc in cell-cell associations during biofilm formation through activity of the protein SasG (Conrady et al., 2008; Formosa-Dague et al., 2016). Additionally, derivatives of the compound 2-aminobenzimidazole (2-ABI) have proven successful in dispersing biofilms formed by methicillin-resistant *S. aureus* (MRSA), vancomycin-resistant *Enterococcus faecium* (VRE), and *Staphylococcus epidermidis* through a zinc-dependent mechanism (Rogers et al., 2009). In this study, we found that similarly to what has been shown in multiple *Staphylococcus* species, *S. pneumoniae* begins to aggregate when exposed to high concentrations of zinc as quickly as 1 h after exposure. Understanding the mechanism responsible for zinc-dependent aggregation in pneumococcus, could present a novel drug target with the ability to inhibit biofilm formation possibly impacting multiple species.

MRSA, VRE, *S. epidermidis*, and *S. pneumoniae* are some of the leading Gram-positive pathogens in terms of both morbidity and mortality, and the ability to form biofilms is an extremely important aspect of pathogenesis for each of these microorganisms. Development of novel therapeutics that could hinder zinc acquisition or aim to block colonization and biofilm formation would drastically help to combat these significant human pathogens. Additionally, given the dramatic increase in antibiotic resistance in these species, specifically, identifying new targets for antimicrobials is imperative.

## Author contributions

LB and RC performed experiments, analyzed data, and wrote the manuscript. TS assisted with data analysis and statistical analyses. EH, VM, and SC assisted with experiments. JR, SS, and JT contributed to experimental design. All authors were involved in editing the manuscript.

### Conflict of interest statement

The authors declare that the research was conducted in the absence of any commercial or financial relationships that could be construed as a potential conflict of interest.

## Supplementary material

Supplementary Figure 1 AdcAII is not involved in biofilm formation. **(A)** Analysis of biofilms formed by the ΔAdcAII strain at 250 and 500 μM zinc using the Mann Whitney *U*-test indicated a *p*-value of < 0.0001. **(B)** Representative growth curves of the ΔAdcAII strain grown in 0–500 μM zinc indicate a growth defect in samples supplemented with 0 and 100 μM zinc.

Supplementary Figure 2 Growth curves of T4R. T4R grown in the presence of **(A)** 0–500 μM zinc, **(B)** 0–45 μM copper, or **(C)** 0–5 μM manganese indicate growth defects present in samples lacking metal supplementation.

Supplementary Figure 3 Viability of *S. pneumoniae* TIGR4 and EF3030 strains. Viability of both **(A)** TIGR4 and **(B)** EF3030 grown in culture tubes with varying concentrations of zinc (0–500 μM). TIGR4 grown in 500 μM zinc showed a significant decrease in viability, *p* < 0.05. Growth curve analysis of **(C)** TIGR4 and **(D)** EF3030 indicate that zinc does not increase growth at 500 μM and is therefore not causing the increase in biofilm density.

Supplementary Figure 4 Zinc does not appear to alter neutrophil abundance in nasopharyngeal tissues. Samples collected from the nasopharynx of animals following bacterial challenge were stained with a hematology neat kit. Light microscopy of tissue samples indicated no significant differences between animals in the 0 and 250 μM treatment groups. Analysis of group means using the Mann Whitney *U*-test revealed a *p*-value of 0.43.

Supplementary Figure 5 Zinc does not appear to protect against pneumococcal H_2_O_2_ in biofilms. **(A)** Biofilms grown by the ΔSpxB strain indicate an increase in density when supplemented with 100–250 μM zinc, and a decrease in biofilm density when supplemented with 500 μM zinc. **(B)** Representative growth curves of the ΔSpxB strain grown in 0–500 μM zinc indicate a growth defect in samples supplemented with 0 and 500 μM zinc. **(C)** Viability plating of biofilms grown in the presence of 0–500 μM zinc.

Supplementary Figure 6 Growth curve of ΔLytA in 0–500 μM zinc. Representative growth curves of the ΔLytA strain grown in 0–500 μM zinc indicate a growth defect in samples supplemented with 0 and 500 μM zinc.

Supplementary Figure 7 Composite biofilm figure of parental T4R, ΔSpxB, and ΔLytA. Biofilms of all strains were grown for 6 h with zinc supplemented from 0 to 500 μM zinc, then rinsed and stained with crystal violet.
