# Supplementary figures and images for: Increased Zinc Availability Enhances Initial Aggregation and Biofilm Formation of Streptococcus pneumoniae
Source: Front Cell Infect Microbiol. 2017 Jun 7;7:233. doi: 10.3389/fcimb.2017.00233 (PMC5461340; doi:10.3389/fcimb.2017.00233)

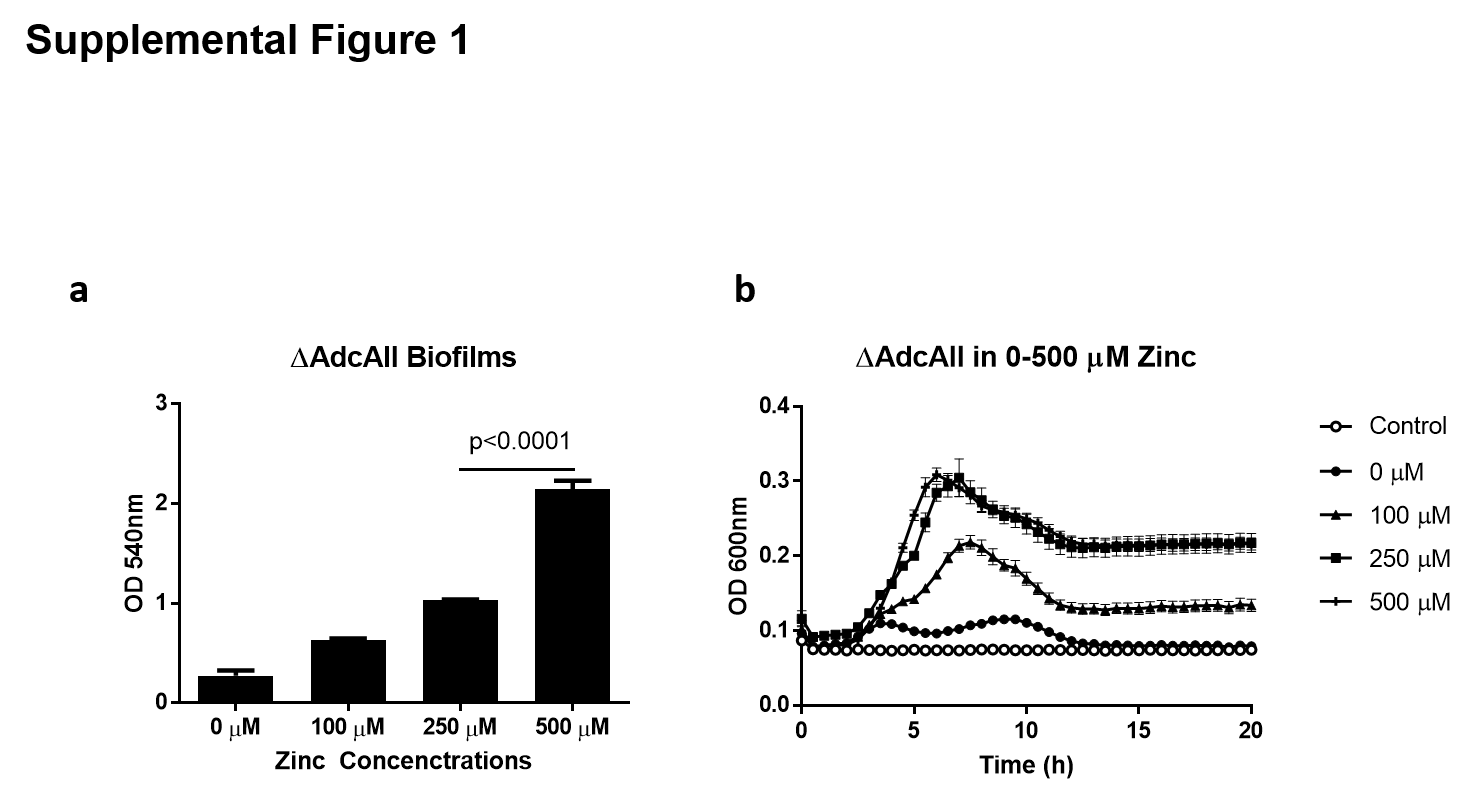

Supplement: Supplementary Figure 1 — AdcAII is not involved in biofilm formation. (A) Analysis of biofilms formed by the ΔAdcAII strain at 250 and 500 μM zinc using the Mann Whitney U-test indicated a p-value of < 0.0001. (B) Representative growth curves of the ΔAdcAII strain grown in 0–500 μM zinc indicate a growth defect in samples supplemented with 0 and 100 μM zinc. [file Image1.TIF]

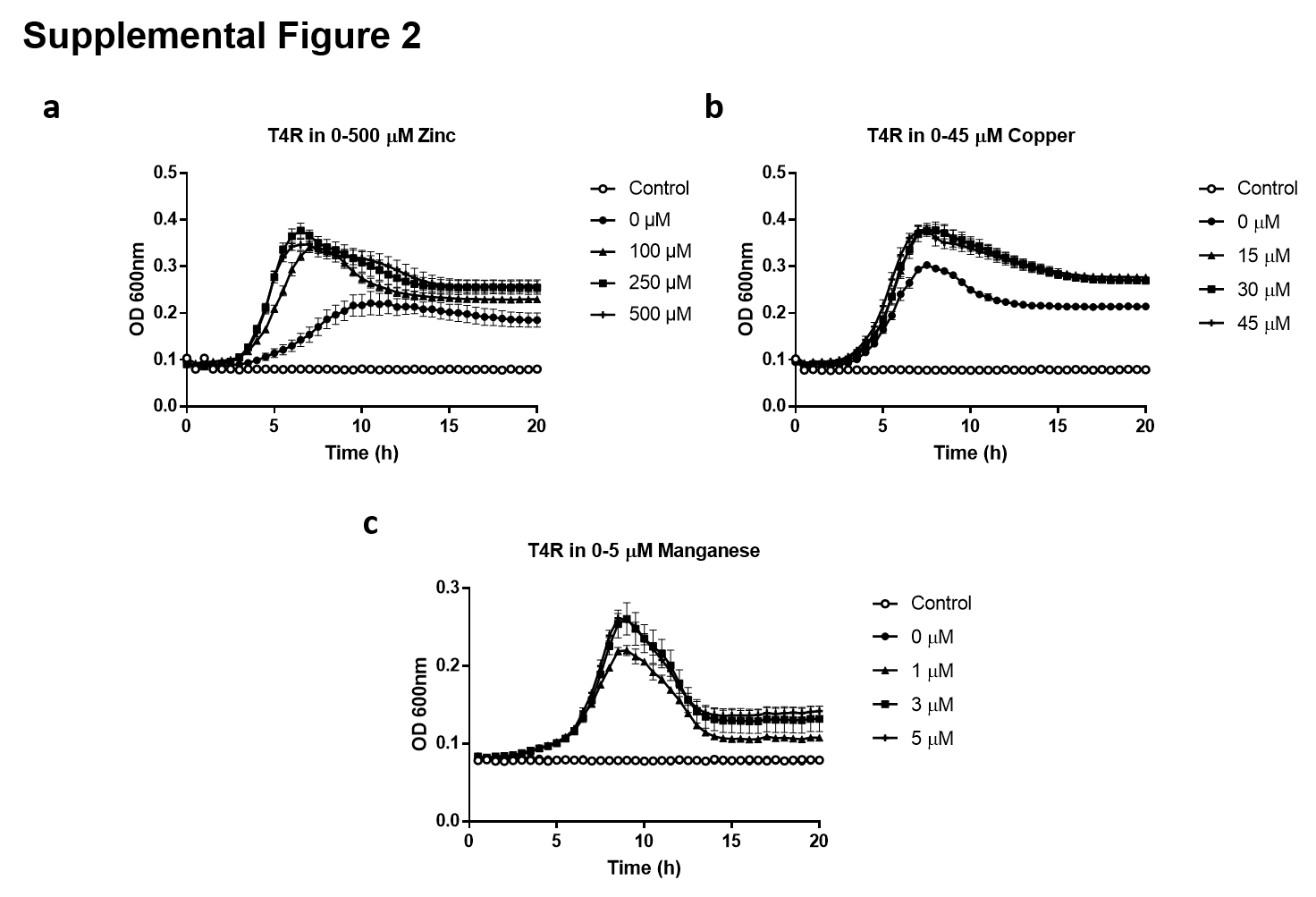

Supplement: Supplementary Figure 2 — Growth curves of T4R. T4R grown in the presence of (A) 0–500 μM zinc, (B) 0–45 μM copper, or (C) 0–5 μM manganese indicate growth defects present in samples lacking metal supplementation. [file Image2.TIF]

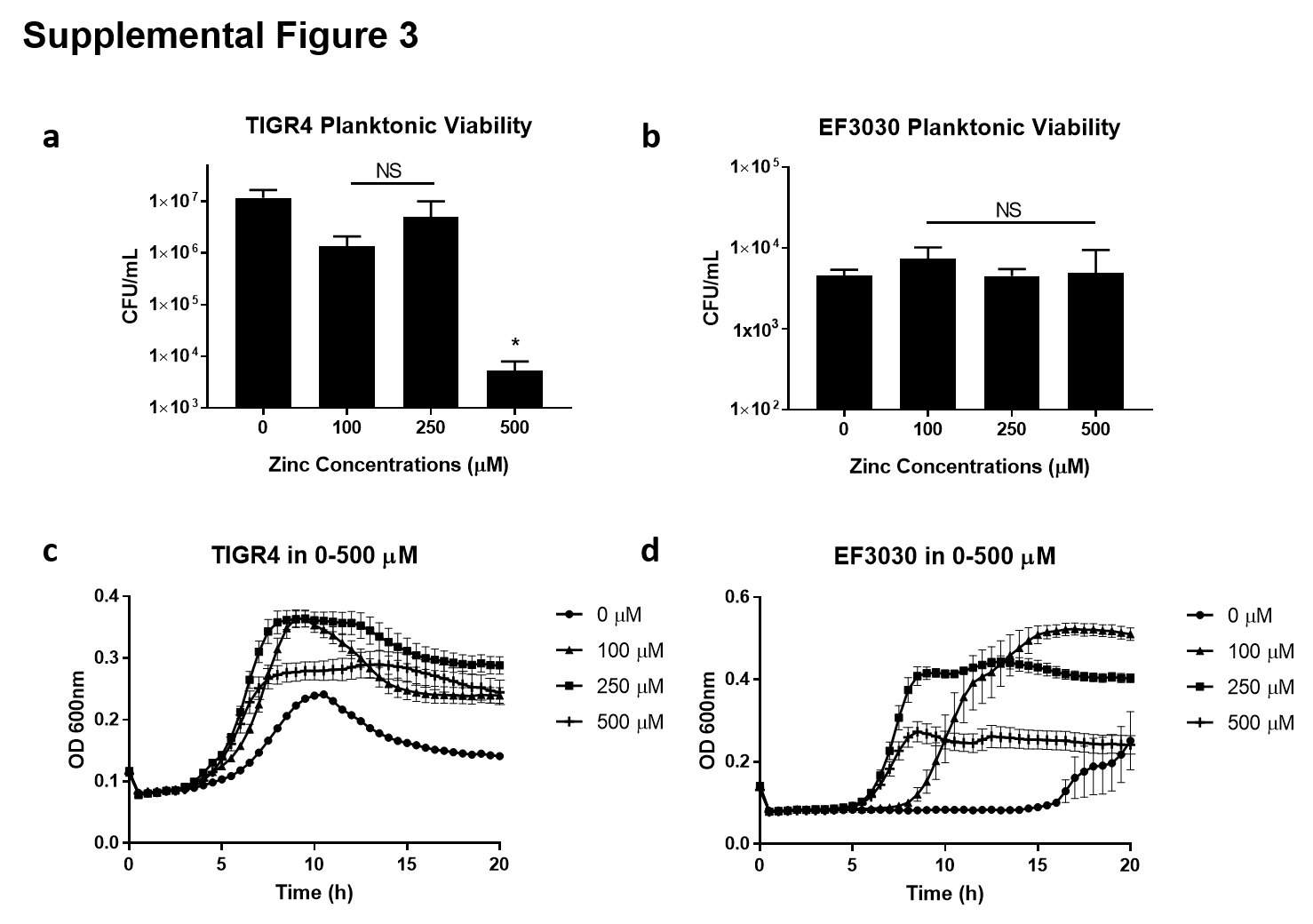

Supplement: Supplementary Figure 3 — Viability of S. pneumoniae TIGR4 and EF3030 strains. Viability of both (A) TIGR4 and (B) EF3030 grown in culture tubes with varying concentrations of zinc (0–500 μM). TIGR4 grown in 500 μM zinc showed a significant decrease in viability, p < 0.05. Growth curve analysis of (C) TIGR4 and (D) EF3030 indicate that zinc does not increase growth at 500 μM and is therefore not causing the increase in biofilm density. [file Image3.TIF]

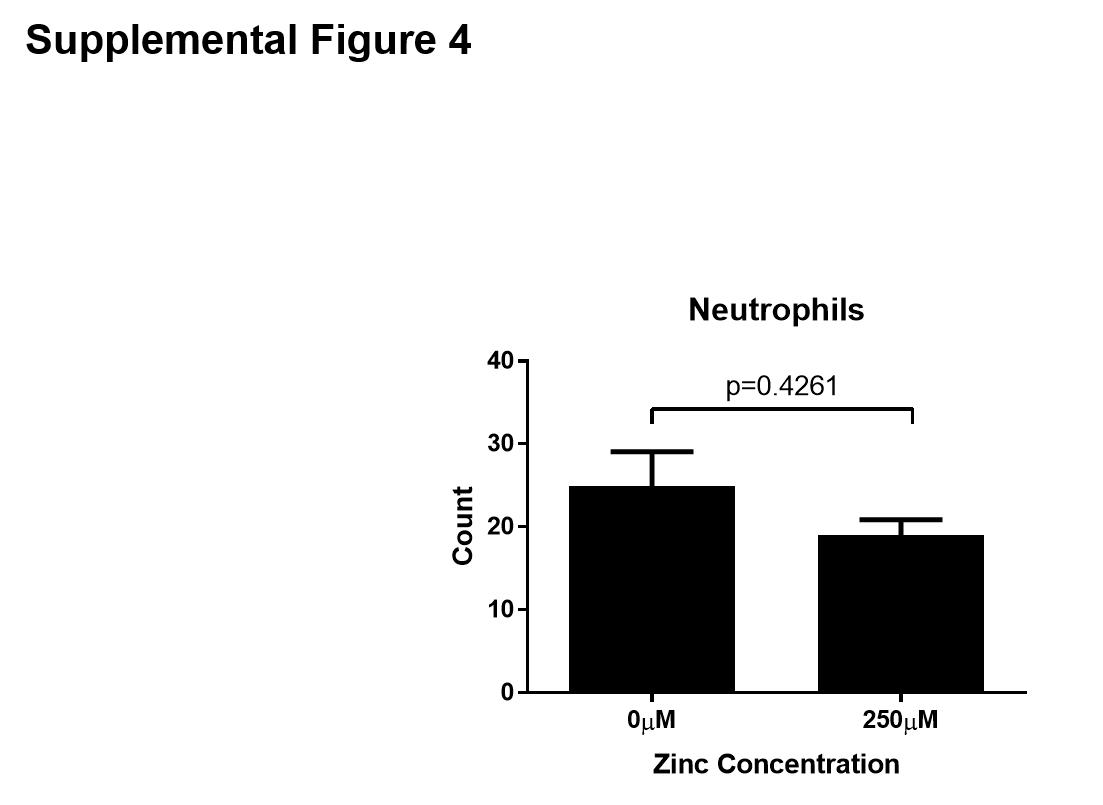

Supplement: Supplementary Figure 4 — Zinc does not appear to alter neutrophil abundance in nasopharyngeal tissues. Samples collected from the nasopharynx of animals following bacterial challenge were stained with a hematology neat kit. Light microscopy of tissue samples indicated no significant differences between animals in the 0 and 250 μM treatment groups. Analysis of group means using the Mann Whitney U-test revealed a p-value of 0.43. [file Image4.TIF]

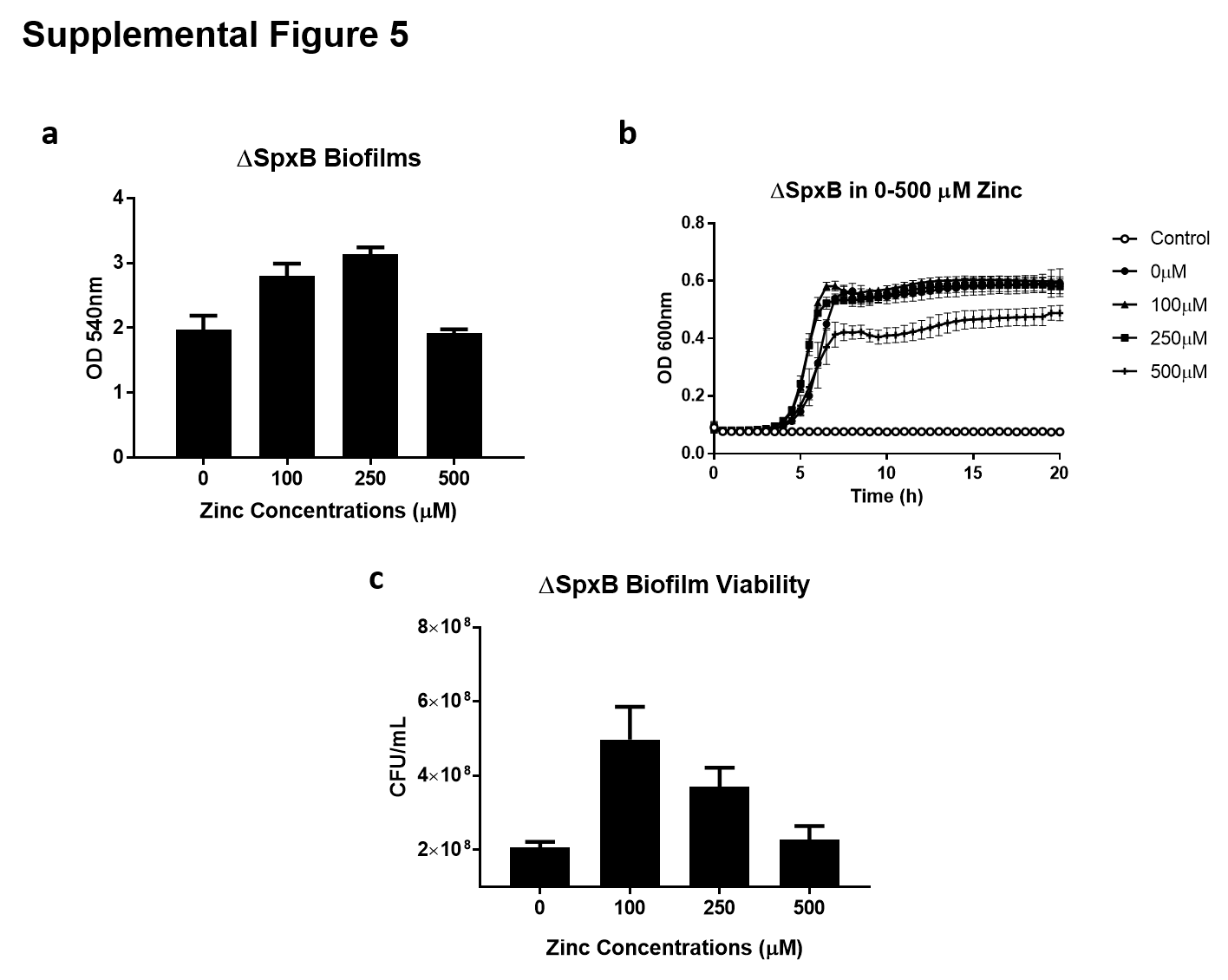

Supplement: Supplementary Figure 5 — Zinc does not appear to protect against pneumococcal H2O2 in biofilms. (A) Biofilms grown by the ΔSpxB strain indicate an increase in density when supplemented with 100–250 μM zinc, and a decrease in biofilm density when supplemented with 500 μM zinc. (B) Representative growth curves of the ΔSpxB strain grown in 0–500 μM zinc indicate a growth defect in samples supplemented with 0 and 500 μM zinc. (C) Viability plating of biofilms grown in the presence of 0–500 μM zinc. [file Image5.TIF]

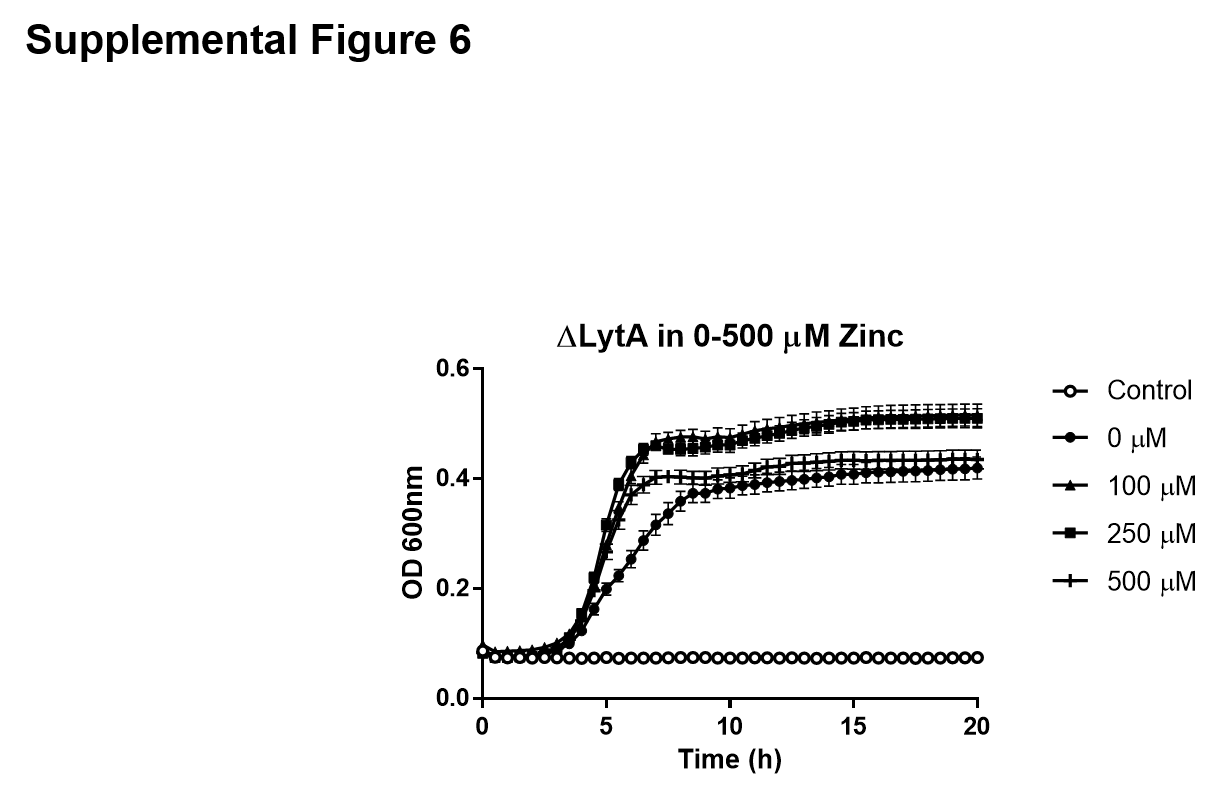

Supplement: Supplementary Figure 6 — Growth curve of ΔLytA in 0–500 μM zinc. Representative growth curves of the ΔLytA strain grown in 0–500 μM zinc indicate a growth defect in samples supplemented with 0 and 500 μM zinc. [file Image6.TIF]

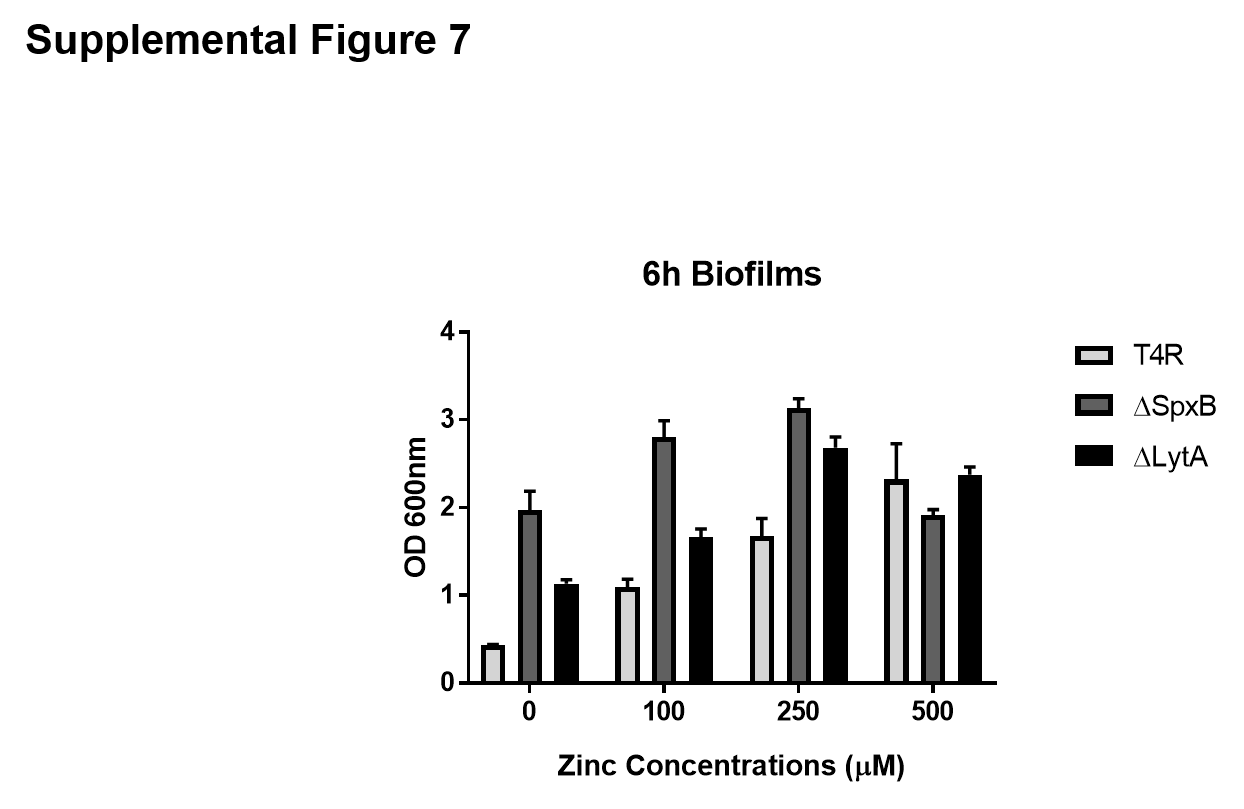

Supplement: Supplementary Figure 7 — Composite biofilm figure of parental T4R, ΔSpxB, and ΔLytA. Biofilms of all strains were grown for 6 h with zinc supplemented from 0 to 500 μM zinc, then rinsed and stained with crystal violet. [file Image7.TIF]
